# Significant Influence of Bound Rubber Thickness on the Rubber Reinforcement Effect

**DOI:** 10.3390/polym15092051

**Published:** 2023-04-26

**Authors:** Jian Chen, Maoyuan Hu, Yuming Li, Rui Li, Long Qing

**Affiliations:** 1 School of Materials Science and Engineering, Sichuan University of Science & Engineering, Zigong 643000, China; 18828872814@163.com (M.H.);; 2 Material Corrosion and Protection Key Laboratory of Sichuan Province, Zigong 643000, China

**Keywords:** carbon black, pyrolysis, natural rubber, bonded rubber, atomic force microscopy

## Abstract

In this work, the contribution of different types of carbon blacks (N115, N330, N550, N660) and their primary and secondary thermally cracked recovered carbon blacks to the mechanical properties of NR composites was evaluated. The thermally cracked recovered carbon blacks were prepared by cracking the rubber composites at 500 °C and de-hybridizing them at 900 °C. The characterization of the thermally cracked recovered carbon blacks by scanning electron microscopy, Raman spectroscopy, and X-ray photoelectron spectroscopy showed that carbon blacks after primary and secondary thermal cracking recovery were more prone to aggregation and exhibited a higher degree of carbon defects. The number and type of functional groups on the surface of these carbon blacks were significantly reduced. For NR composites with pristine samples added, the mechanical properties and the bound rubber content tests showed that the mechanical properties of the NR composites became weaker with the increase in carbon black particle size. The bound rubber content also decreased with increased carbon black particle size. The mechanical properties of the NR composites reinforced with carbon black recovered by primary and secondary thermal cracking would therefore decrease. The results of AFM and DSC tests further confirmed the decreasing trend of bound rubber. The present work demonstrates the effect of bound rubber content variation on the mechanical properties of rubber, demonstrates the morphology of bound rubber more visually, and provides new insights into the reinforcement theory of CB.

## 1. Introduction

As an important industrial raw material, carbon black (CB) has many applications in the rubber, plastic, and coating industries [1,2,3,4]. Surveys have shown that the CB produced as a rubber pigment, reinforcing agent, thermal conductivity additive, and used in automotive tires accounts for 70% of the total. CB is a black powder filler [5,6], and this black powder material is mainly generated by various gas-phase hydrocarbon substances through thermal cracking reaction or incomplete combustion. Its chemical element is C [7,8,9,10]. The observation of CB by electron microscopy noted many microcrystal structures in CB. Carbon atoms are closely arranged due to the interaction between carbon atoms [11,12,13]. The carbon atoms in CB usually form microcrystalline structures in these layers. Therefore, external forces do not easily destroy these microcrystalline structures due to the high-strength bonding between the chemical bonds [14,15,16]. CB is an important reinforcing filler. In the rubber industry, adding CB to the rubber matrix will significantly improve the performance of rubber products [17,18,19,20]. It can not only improve rubber’s tensile and break strength but also improve rubber’s wear resistance and thus extend rubber’s service life; so far, for more than 100 years [21,22].

The reinforcing effect of CB is mainly reflected by the particle size (specific surface area), structure, surface chemical activity, etc. The macroscopic manifestation is the formation of bound rubber, also called CB gel, refers to the part of the rubber in the filled unvulcanized compounding rubber that cannot be dissolved by a normally effective solvent [23,24]. In essence, it is the substance adsorbed onto the surface of the filler, i.e., the rubber in the interfacial layer between the filler and the rubber, which has characteristics similar to the glass state. The bound rubber is strong if there is more bound rubber, so the bound rubber is the yardstick for measuring the reinforcing ability of CB [25,26,27]. There are two reasons for the generation of bound rubber: (1) the molecular rubber chains adsorbed onto the surface of CB combine with the surface groups of CB; or (2) the rubber generates a large number of rubber radicals or ions that combine with CB after mixing and vulcanization during processing. Chemical adsorption occurs, which is the main way that bound rubber is generated [28,29,30]. Those macromolecular rubber chains on the surface of CB particles are physically adsorbed with greater solubility. It is difficult to unbind all the macromolecular chains adsorbed by CB simultaneously. As long as one or two adsorbed links are not removed, the whole molecular chain can become bonded rubber.

In addition, the recycling of waste tires has also become one of the research hotspots, and scientific researchers often recover CB by the thermal cracking of waste tires [31,32]. However, chemical bonds are formed between certain rubber polymer chains and CB surfaces during the rubber refining process. Rubber thermal cracking of CB and rubber for blending forms a layer of bonding rubber, and this bound rubber layer after thermal cracking treatment will be in a certain glassy state. To a certain extent, coking and ash deposition occur, making the high-temperature cracking of scrap tires unable to completely remove the bound rubber [33,34,35,36]. 

In this work, we aim to simulate the thermal cracking process of tires, prepare an N-series of rubber thermally cracked CBs and an N-series of rubber secondary thermally cracked CBs with N-series CBs, study the bonding rubber formed between them and the rubber, examine the relationship between the bonding rubber content and the thickness of the bonding rubber based on the N-series CBs, and try to determine a certain law. We also try to determine the effect of the thermal cracking of CB on the binding gel content and the particle size. Through the study of rubber thermal cracking of CB, the influence of thermal cracking behavior on the bound rubber is shown, which provides a theoretical basis for tire cracking and the utilization of the resulting tire-cracked CB and provides new insights into the theory of CB reinforcement.

## 2. Experimental Section

### 2.1. Materials

Natural rubber (NR), CBs (N115, N330, N550, N660), stearic acid (SA), zinc oxide (ZnO), sulphur, 2,2-dibenzoth-iazoledisulfde (DM) were all commercial grade and were obtained from China Car-bon Black Institute (Zigong, China). Dimethyl benzene (purity: 99.5%) and acetone (purity: 99.0%) were obtained from Kelong Chemical Reagent Co., Ltd. (Chengdu, China)

### 2.2. Preparation of NR/CB Composites

The NR/CB composites (NR (100 phr), CB (50 phr, N115/N330/N550/N660), S (2.5 phr), SA (3 phr) and DM (0.6 phr) were prepared on a two-roll mill with the formula shown in Table 1. Briefly, the process is as follows: Firstly, heat and preheat the opener to about 70 °C and break the natural rubber without wrapping the roll. Then, adjust the whole roll distance to wrap the natural rubber in the front roll, add the other ingredients for mixing to make the blended rubber, and vulcanize it at 145 °C for 30 min after placing it on a smooth platform for 24 h to obtain the NR/CB composites. These rubber composites with different series of CB additions are named NR/N115a, NR/N330a, NR/N550a, NR/N660a.

### 2.3. Preparation of Recycled CBs and Their Corresponding Rubber Composites

The rubber composites prepared in Section 2.2 were thermally cracked at 500 °C using a tube furnace and then de-hybridized at 900 °C to produce an N-series of rubber primary thermally cracked CBs, named N115b, N330b, N550b, N660b, respectively (the original samples were named N115a, N330a, N550a, N660a, respectively). Then, these primary thermally cracked CBs were used to fill natural rubber according to the formula shown in Table 1, and natural rubber composites were obtained, named NR/CB-115b, NR/CB-330b, NR/CB-550b, NR/CB-660b, respectively. These primary thermally cracked CBs were then used to fill natural rubber to obtain natural rubber composites named NR/N115b, NR/N330b, NR/N550b, NR/N660b.

The above process was repeated to obtain the secondary thermally cracked CBs named N115c, N330c, N550c, N660c, and their corresponding rubber composites, named NR/N115c, NR/N330c, NR/N550c, NR/N660c, respectively.

### 2.4. Characterization

The rubber composites were cut using an ultra-thin cryogenic slicer (Leica EM UC7, Leica Microsystems (Shanghai) Trading Co., Ltd., Shanghai, China) to obtain flat surfaces and then scanned with an atomic force microscope (AFM, E-SWEEP, Hitachi High-Tech Technology Co., Ltd., Tokyo, Japan). The microscopic morphology of the samples was examined using a scanning electron microscope (SEM, Hitachi S4800, Tokyo, Japan). The X-ray diffraction (XRD, D2, Bruker., Saarbrucken, Germany) analysis was performed using a diffractometer. X-ray photoelectron spectroscopy (XPS) analysis was performed on an Escalab 250 XPS system (Thermo Electron Corporation, Washington, DC, USA) with an Al Kα X-ray source (1486.6 eV). A Cu Kα diffraction source (λ = 0.154 nm) was used, and Raman spectroscopy measurements were performed using a confocal Raman spectrometer (Renishaw inVia, London, UK) with a laser wavelength of 633 nm. Thermogravimetric analysis (Q500, TA Corporation, Washington, DC, USA) was performed under air atmosphere from RT to 800 °C with 10 °C/min. Fourier transform infrared (FT-IR, Bruker Vertex 70; Saarbrucken, Germany) spectroscopy of KBr slices was performed at 400~4000 cm^−1^. The calorimetric glass transition process was studied by differential scanning calorimeter (DSC, TA, Washington, USA) and used for dynamic mechanical analysis with a measurement temperature range of −70 °C to 60 °C and a ramp rate of 5 °C/min.

The mechanical properties of the rubber for blending were tested by a tensile testing machine (RGM-50, Shanghai, China) at a tensile rate of 500 mm/min. The hardness of the rubber was tested using a hardness tester (JLX-A, Guangzhou, China) of type shore A. The dynamic mechanical properties of SBR composites were tested by a dynamic mechanical testing method using the Dynamic mechanical analyzer (DMA, Q800, Washington, DC, USA) at a temperature range of −100–80 °C. The bound rubber content was determined by the equilibrium expansion method, which is described in the supporting information [12]. 

## 3. Results and Discussion

### 3.1. Characterization of the Recycled CBs

**Figure 1:**
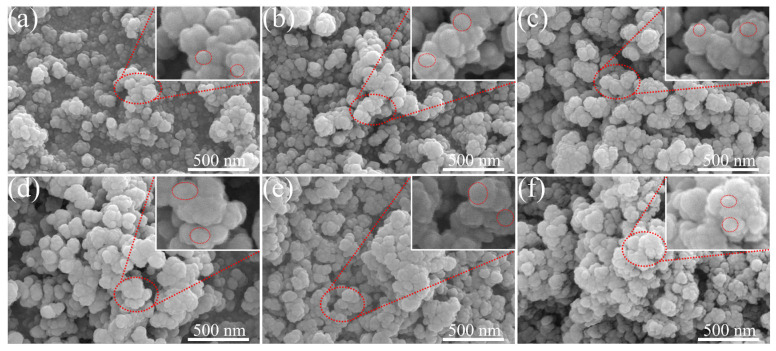
SEM image of (**a**) N115a, (**b**) N330a, (**c**) N550a, (**d**) N660a, (**e**) N330b, (**f**) N330c.

Figure 1a–d show the microscopic morphology of the N-series carbon blacks. It can be seen that the carbon black particle size gradually increases with the model, with N330a having the smallest particle size and N660a having the largest particle size, which is consistent with the relevant reports from the conventional industry [37,38]. Figure 1e,f then display the microscopic morphology of carbon black after the first and second thermal cracking recovery of N330. Although N330b and N330c have similar morphology to N330a (pure sample), the boundary between the particles of N330a is obvious. In contrast, N330b and N330c show a rare agglomeration phenomenon, sticking to each other with a significantly higher confusion than N330a, which may be because the rubber composites were subjected to thermal decomposition and the combined effect of residual tar and bound rubber [3,39]. This phenomenon also occurred in the corresponding thermally cracked recovered carbon blacks of N115a, N550a, and N660a (Figure S1). Figure S2b presents the SEM view of NR/N330a. NR/N330a is not smooth because ZnO and CB nanoparticles are attached to the surface of NR/N330a. The elemental profiles demonstrate that C, O, Zn, and S are uniformly represented in NR/N330a. In addition, the SEM–EDS mapping of NR/N115a, NR/N550a, and NR/N660a display similar SEM–EDS mapping results, as shown in Figure S2.

**Figure 2:**
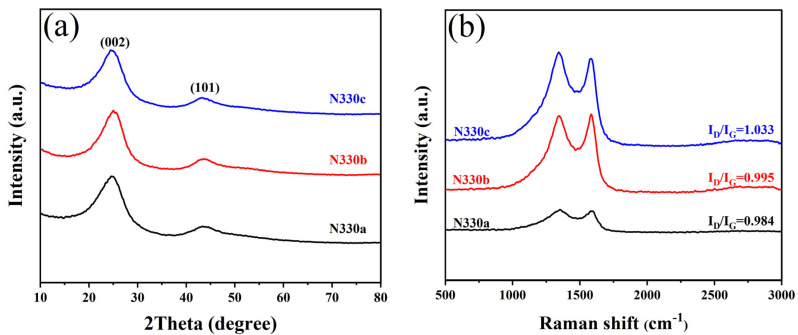
(**a**) XRD patterns and (**b**) Raman spectrums of N330a, N330b, N330c.

The XRD spectra of the samples are shown in Figure 2a. All samples have two flat and broad diffraction peaks with diffraction angles around 24° and 43°, representing the (002) and (101) diffraction surfaces, respectively, indicating a disordered structure [40]. The intensity of the three peaks was relatively close to each other without significant differences, indicating that the impurities were removed during the first and second thermal cracking of the recovered carbon black [41]. The samples N115a, N550a, and N660a showed the same trend (Figure S3).

The samples were further structurally characterized by Raman spectroscopy. As seen in Figure 2b, all materials show two distinct broad peaks corresponding to the D peak (1343 cm^−1^) and the G peak (1580 cm^−1^), indicating a typical amorphous carbon structure [42,43]. The intensity ratios (I_D_/I_G_) of the D and G peaks are usually used to estimate the defect degree of graphene. The I_D_/I_G_ values of N330a, N330b, and N330c are 0.984, 0.995, and 1.033, respectively, indicating that the carbon blacks recovered after thermal cracking exhibit a lower defect degree, which is related to the chemical reactions occurring on the carbon black surface during the pyrolysis process. In addition, Raman tests were also performed on the samples of N115a, N550a, and N660a. The results showed that N115a, N550a, and N660a exhibited lower defects after the first and second thermal cracking recovery (Figure S4).

**Figure 3:**
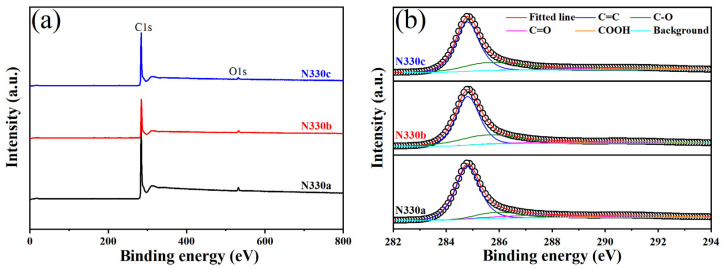
(**a**) XPS full spectra and (**b**) C1s spectra of N330a, N330b, N330c.

The chemical composition and chemical state were tested by Xray photoelectron spectroscopy (XPS). Figure 3a shows the full spectrum analysis of N330a, N330b, and N330c. The exact elemental content is shown in Table 2. For the pure sample N330a, the C and O element percentages were 97.16% and 2.46%, respectively, consistent with previously reported literature [24]. However, for the N330b recovered by the first thermal cracking, the content of C and O elements were 95.44% and 3.02%, respectively, and even contained 0.95% of the N element. This change in elemental content must be caused by the interaction between N330b and the rubber matrix and other additives and the vulcanization reaction. Figure 3b shows the C1s fine spectra of N330a, N330b, and N330c, all of which can be seen as C=C/C-C (284.8 eV), C-O (285.8 eV), C=O (287.0 eV), O-C=O (290.9 eV) [44]; the difference lies in the different contents, as shown in Table 2. The content of C=O bonds in N330a, N330b, and N330c is 10.49%, 5.09%, and 3.22%, respectively, showing a gradual decrease. The content of C=C/C-C bonds in N330a, N330b, and N330c is 67.70%, 58.72%, and 63.06%, respectively, showing a downward trend. To further illustrate, the FTIR spectra of the three were tested. As shown in Figure S5, the C=O bond of N330b was almost not shown in the FTIR spectra. In the XPS, the samples N115a, N550a, and N660a showed the same trend, where the number of functional groups decreased.

### 3.2. Properties of Rubber Composites

**Figure 4:**
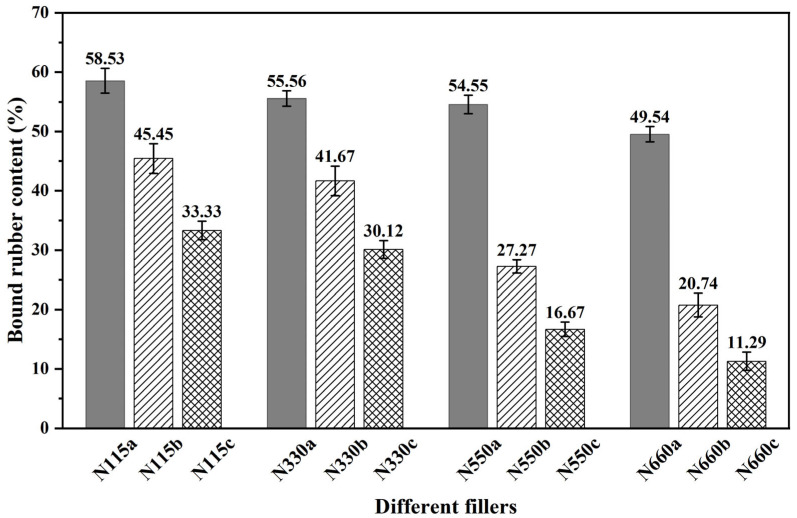
Effect of different fillers on the bound rubber content.

The bound rubber content is strong evidence of the filler’s effect on rubber reinforcement [3]. As shown in Figure 4, the measured binding gel content of rubber composites filled with N115a, N330a, N550a, and N660a and their primary and secondary thermal cracking recovered carbon black is demonstrated. It can be seen that for the rubber composites with pure samples N115a, N330a, N550a, and N660a added, the bonded rubber content gradually decreases from 58.53% at the beginning for NR/N115a to 49.54% for NR/N660a. This indicates that the smaller the particle size of carbon black, the more bonded rubber content is formed. In addition, taking NR/N330a, NR/N330b, and NR/N330c as examples, N330a also showed a gradual decrease in the binding gel content after primary and secondary thermal cracking recovery, which may be related to the decrease in the number and type of functional groups on the carbon black surface [45,46]. This is also consistent with the results of the previous XPS analysis.

**Figure 5:**
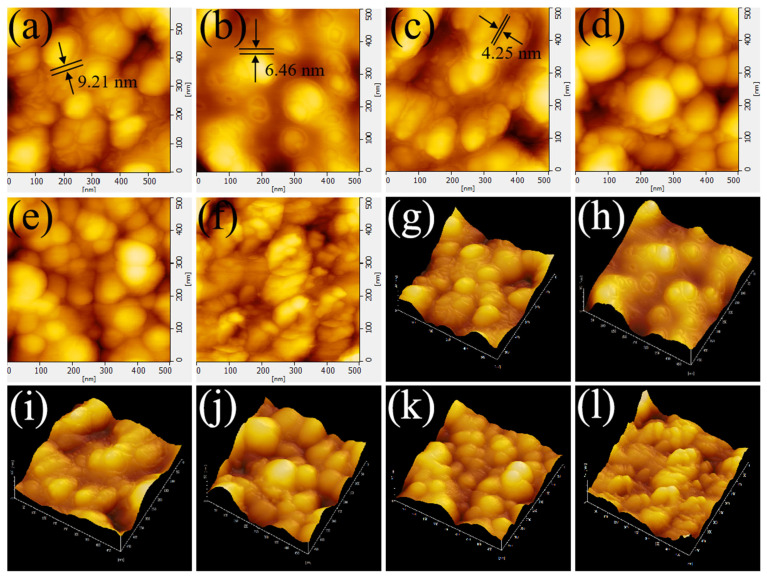
AFM micrographs of (**a**) NR/N115a, (**b**) NR/N330a, (**c**) NR/N550a, (**d**) NR/N-660a, (**e**) NR/N115b, (**f**) NR/N330b; and the 3D maps of (**g**) NR/N115a, (**h**) NR/N330a, (**i**) NR/N550a, (**j**) NR/N-660a, (**k**) NR/N115b, (**l**) NR/N330b.

To further observe the distribution of the rubber filler and the binding gel’s state on the carbon black surface, the frozen section samples were observed using AFM, as shown in Figure 5a–d. Surprisingly, for the NR/N115a and NR/N330a composites, it was observed from the morphology that the surface of the black carbon particles was wrapped with a layer of binding gel. Due to the high hardness of the carbon black nanoparticles, the cutting knife cut through the binding gel on the surface of the carbon black when the samples were prepared using frozen ultra-thin sections, thus leaving the carbon black exposed. However, for the NR/N550a composite, the bonding adhesive covered by the N550a surface was not obvious. In the NR/N660a composite, the surface of N660a was so smooth that no visible bonding rubber coating was even observed, and the results observed by AFM tended to be consistent with the bonding rubber content test results. This may be due to factors such as the difference in particle size and specific surface area of different series of carbon blacks. In addition, as shown in Figure 5e,f, the morphology of the bound rubber in the NR/N115b and NR/N330b composites was also observed by AFM. However, it was difficult to observe the bound rubber coating layer on the surface of N115b and N330b, i.e., it was difficult to form the bound rubber in the rubber composites filled with N115a and N330a after the first thermal cracking recovery [47], which is consistent with the results of the bound rubber content test. This is confirmed by the decrease in the bonding adhesive content of NR/N115a and NR/N330a in the test results. In addition, the thickness of the bound rubber of NR/115a, NR/N330a, and NR/N550a were fitted and measured (Figure S6). The specific thickness is shown in Table 3.

**Figure 6:**
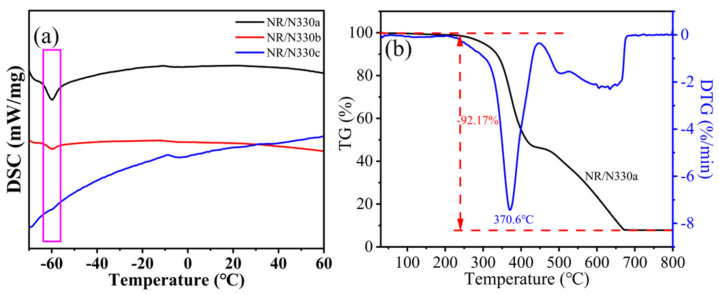
(**a**) DSC curves of NR/N330, and (**b**) TG–DTG curves of NR/N330a.

Figure 6a shows the DSC curves of NR/N330a, NR/N330b, and NR/N330c rubber composites. The DSC curve of NR/N330a composites shows an obvious inflection point in the range of −62~−58 °C, which is a typical sign of glass transition [48,49]. NR/N330b composites showed a similar but weaker change over the same temperature range, while NR/N330c showed almost no such change, which was related to the gradual reduction of binding adhesive content in NR/N330a, NR/N330b, and NR/N330c composites. It may contain a small amount of NR rubber that has not been dissolved by toluene, making its DSC curve show a weak inflection point. In contrast, NR/N330c composites have even less bound rubber. The unbound NR rubber was completely dissolved by toluene, so the NR/N330c composite did not show any change in the temperature range tested. This once again proves that N330a has lower bound rubber content after the primary and secondary thermal cracking recovery to form NR/N330c composites [50]. In addition, the trend of DSC curves of NR/N115a, NR/N550a, and NR/N660a was similar, as shown in Figure S7.

N115a, N330a, N550a, N660a, and their primary and secondary thermal cracking recovered carbon blacks with the mechanical properties of the NR composites. The results are shown in Table 4. It was learned from the previous SEM tests that the particle sizes of N115a, N330a, N550a, and N660a gradually increased. The NR/N115a composites showed the best tensile strength and elongation at break when N115a, N330a, N550a, or N660a were added to the NR matrix. With the increase in carbon black particle size, the tensile strength and elongation at break of NR composites gradually decreased [6,7]. The tensile strength finally decreased from 25.26 MPa to 19.95 MPa. This is consistent with the conclusion of previous studies that the smaller the carbon black particle size, the better the reinforcement effect. In addition, the bound rubber content and AFM tests revealed that the NR/N115a composites had the highest bound rubber content, which again proved that the smaller the carbon black particle size, the higher the surface bound rubber content [51,52], substantially improving the mechanical properties of the NR composites.

Figure 6b shows the thermogravimetric and differential thermal gravity curves of NR/N330a at air atmosphere in the range of 30 to 800 °C. As can be seen, the residual rubber hydrocarbon content in NR/N330a gradually decreases with the increase in temperature, and the biggest weight loss occurs at about 370.6 °C [35]. Beyond 670 °C, TG and DTG curves become flat, and the mass no longer changes. In addition, the TG–DTG of NR/N115a, NR/N550a, and NR/N660a showed similar TG–DTG curve trends, as shown in Figure S8.

Table 4 shows the mechanical properties of NR composites. Further tests revealed that the mechanical properties of the NR composites were significantly reduced after using both primary and secondary thermally cracked recycled carbon blacks to reinforce the NR composites. For example, the tensile strength and elongation at break of NR/330a composites were 24.18 MPa and 448.19%, respectively. In comparison, the tensile strength and elongation at break of NR/330b composites were reduced to 13.64 MPa and 369.05%, respectively. The modulus at 300% elongation and hardness of NR/330a composites were 16.28 MPa and 72.80, respectively. In comparison, the tensile strength and hardness of NR/330c composites were reduced to 5.01 MPa and 60.50, respectively. This may be due to the co-action of NR matrix and carbon black in the process of thermal cracking. The carbon black recovered from thermal cracking is aggregated, which changes the size of the carbon black aggregate, the degree of surface defects, and the number and type of functional groups. As a result, the binder content of NR is reduced when it is reinforced again, and the mechanical properties of NR show obvious attenuation at the macro scale [43].

**Figure 7:**
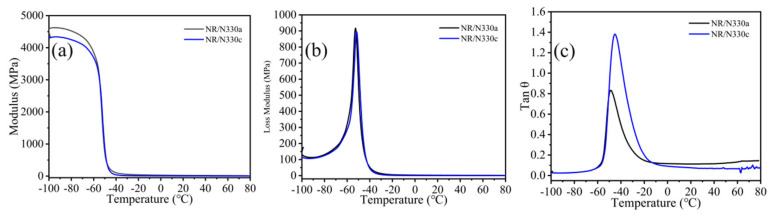
(**a**) Storage modulus versus temperature curve of NR/N330, (**b**) loss modulus versus temperature curve of NR/N330, and (**c**) loss factor versus temperature curve of NR/N330.

Figure 7a–c show the storage modulus (E′), the loss modulus (E′′), and the tan θ-temperature curves of the NR composites. E′ is proportional to the maximum elasticity stored in the rubber sample in each cycle, reflecting the elastic component in the viscoelastic rubber. E′′ is related to the energy consumed by the rubber sample in each cycle, reflecting the viscosity component in the rubber viscoelasticity [44,45,46]. The internal friction value (tan θ) at 0 °C is used to evaluate the wet-skid resistance, while tan θ at 60 °C represents the rolling resistance [18,47]. As shown in Figure 7a, the E′ of the NR/N330a composite is higher than that of the NR/N330c composite. The result reveals that NR/N330c reduces the vulcanized stored energy and resilience at low temperature, which is adverse for rubber’s safety in some extreme weather condition [48]. As the temperature rises, the rubber segments begin to become soft, and the rubber rigidity decreases gradually, resulting in the decline of E′ [49,51]. With the increase in temperature, the E′ values of NR/N330c and NR/N330a tend to be consistent. In addition, the E′ of NR/N115, NR/N550, and NR/N660 showed similar E′ curve trends, as shown in Figure S9.

Figure 7b shows the loss modulus versus temperature curve of NR/N330. As is well known, a higher E′′ means a larger viscosity deformation and bigger contact area, thereby enhancing the driving safety of vehicles [17,53,54]. The E′ values of NR/N330c and NR/N330a tend to be consistent. The E′′ of NR/N115, NR/N550, and NR/N660 also showed similar E′′ curve trends, as shown in Figure S10.

Figure 7c shows the tan θ–temperature curves of NR composites. The tan θ value of NR/N330a at 0 °C is 0.1161, surpassing that of NR/N330c (0.09116). The tan θ value of NR/N330a at 60 °C is 0.1326, surpassing that of NR/N330c (0.06539). Moreover, the tan θ of NR/N115, NR/N550, and NR/N660 showed similar tan θ–temperature curve trends, as shown in Figure S11. The BET surface area of N330b was lower than that of N330a [55]. The presence of the carbonaceous deposits from the polymer decomposition reduces the surface roughness of RCB and smoothens the surface, resulting in a lower surface area to that N330 [55]. The thermally cracked recovered carbon blacks have a wide pore size distribution, which can be attributed to the mesoporous structure and the effect of large-chain agglomeration [56]. The average calculated pore size was 20–40 Å [57].

## 4. Conclusions

In conclusion, thermally cracked recovered carbon black was prepared by cracking the rubber composite at 500 °C and de-hybridizing it at 900 °C. The test results showed that the degree of carbon defects and the type and number of functional groups on the surface of the thermally cracked recovered carbon black changed. The particle size did change significantly. The mechanical properties and bonding rubber content tests showed that the mechanical properties of NR composites became weaker with the increase in carbon black particle size. The bonding rubber content also decreased with the increased carbon black particle size. The mechanical properties of NR composites reinforced with primary and secondary thermal cracking recovered carbon black would therefore decrease. The bonding rubber content would gradually decrease, and the AFM and DSC tests confirmed the decrease of bonding rubber. The AFM and DSC test results further confirmed the reduction in bonding rubber. In DMA, the thermally cracked recovered carbon black reduces the vulcanized stored energy and resilience at low temperature, which is adverse for rubber’s safety in some extreme weather conditions. This study provides a new idea to explore the theory of reinforcement of rubber composites by nanofillers.

## Figures and Tables

**Table 1:** Formulae of the NR/CB composites.

Samples	phr
NR	100
CB (N115/N330/N550/N660)	50
S	2.5
SA	3
DM	0.6
ZnO	5

**Table 2:** The content of elements and different carbon species in N330a, N330b and N330c.

	Materials	C	O	S	N	C=C/C-C	C-O	C=O	COOH
Content (%) Elements									
N330a		97.16	2.46	0.38	-	67.70	13.99	10.49	7.82
N330b		95.44	3.02	0.59	0.95	58.72	27.95	5.09	8.23
N330c		97.37	2.22	0.41	-	63.06	27.23	3.22	6.49

**Table 3:** The thickness of the bound rubber of NR/115a, NR/N330a, and NR/N550a.

Samples	NR/115a	NR/N330a	NR/N550a
Thickness	7–9 nm	5–6 nm	2–4 nm

**Table 4:** Mechanical properties of NR composites.

Samples	Tensile Strength (MPa)	Elongation at Break (%)	Modulus at 300% Elongation (MPa)	Hardness (Shore A)
NR/N115a	25.26 ± 0.50	471.90 ± 10.00	17.29 ± 0.50	74.20 ± 0.50
NR/N115b	15.68 ± 0.40	371.73 ± 8.00	12.23 ± 0.50	62.80 ± 0.50
NR/N115c	9.60 ± 0.40	295.94 ± 8.00	9.17 ± 0.30	60.20 ± 0.50
NR/N330a	24.18 ± 0.50	448.19 ± 10.00	16.28 ± 0.50	72.80 ± 0.50
NR/N330b	13.64 ± 0.40	369.05 ± 8.00	10.81 ± 0.30	64.50 ± 0.50
NR/N330c	5.02 ± 0.50	267.79 ± 5.00	5.01 ± 0.30	60.50 ± 0.30
NR/N550a	21.02 ± 0.50	434.10 ± 10.00	16.12 ± 0.50	66.20 ± 0.50
NR/N550b	13.55 ± 0.40	351.02 ± 5.00	11.31 ± 0.40	62.40 ± 0.50
NR/N550c	5.47 ± 0.40	260.78 ± 5.00	5.46 ± 0.50	62.20 ± 0.50
NR/N660a	19.95 ± 0.30	418.31 ± 10.00	11.28 ± 0.40	64.80 ± 0.50
NR/N660b	12.37 ± 0.50	332.12 ± 5.00	11.01 ± 0.50	62.10 ± 0.50
NR/N660c	7.98 ± 0.30	251.22 ± 5.00	7.41 ± 0.50	54.80 ± 0.50

## Data Availability

Not applicable.

## Supplementary Materials

The following supporting information can be downloaded at: https://www.mdpi.com/article/10.3390/polym15092051/s1, Scheme S1: A schematic diagram of the fabrication process of NR/N330; Figure S1: SEM image of (a) N115b, (b) N550b, (c) N660b, (d) N115c (e) N550c, (f) N660c; Figure S2: SEM-EDS images of (a) NR/N115a, (b) NR/330A, (c) NR/N550a, and (d) NR/N660a; Figure S3: (a) XRD patterns of (a) N115a, N115b and N115c. (b) N550a, N550b and N550c. (c) N660a, N660b and N660c; Figure S4: (a) Raman spectrums of (a) N115a, N115b and N115c. (b) N550a, N550b and N550c. (c) N660a, N660b and N660c; Figure S5: FTIR spectra of N330a, N330b and N330c; Figure S6: Fitting measurement of NR/115a, NR/N330a and NR/N550a bonding rubber; Figure S7: DSC curves of the (a) NR/N115, (b) NR/N550 and (c) NR/N660; Figure S8: TG-DTG curves of the (a) NR/115a, (b) NR/550a and (c) NR/660a from 30 to 800 °C; Figure S9: Storage modulus versus temperature curve of the (a) NR/115, (b) NR/550 and (c) NR/660 from −100 to 80 °C; Figure S10: Loss modulus versus temperature curve of the (a) NR/115, (b) NR/550 and (c) NR/660 from −100 to 80 °C; Figure S11: Loss factor versus temperature curve of the (a) NR/115, (b) NR/550 and (c) NR/660 from −100 to 80 °C; Figure S12: TG curves of the (a) N330a, and (b) N330c from 30 to 800 °C, with 10 °C/min and at the maximum temperature for 30 min.
