# Supplementary material for: Significant Influence of Bound Rubber Thickness on the Rubber Reinforcement Effect
Source: Polymers (Basel). 2023 Apr 26;15(9):2051. doi: 10.3390/polym15092051 (PMC10181254; doi:10.3390/polym15092051)
Supplement: Supplementary file 1 [file polymers-15-02051-s001.zip › polymers-2276860-supplementary.pdf]

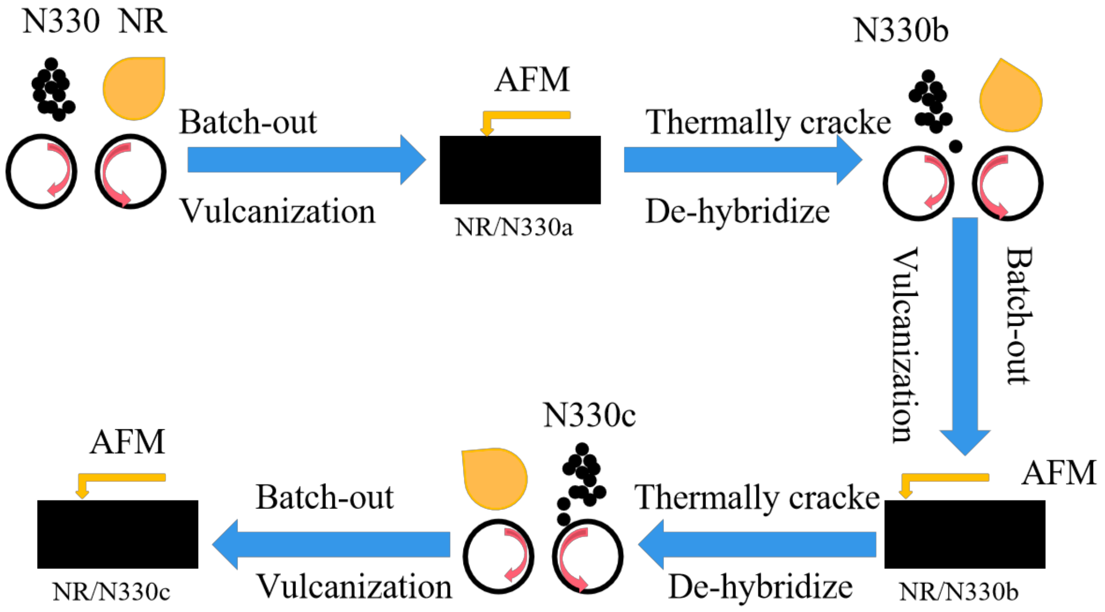

Scheme S1. A schematic diagram of the fabrication process of NR/N330.

The specific testing procedure was as follows, the unvulcanized NR/CB composites were cut into small pieces of about 1mm^3, weighed about 0.5g (W1) and tightly sealed in a clean stainless-steel mesh of consistent weight (W_2). Subsequently, immersed in 100ml toluene for 48h at room temperature, re-solvent changed in the immersion for 24h. Ultimately, this stainless steel meshes were dried to a constant weight (W_3). The bonding rubber rate (R_B%) was calculated by the following equation.

$$Bound\ rubber\ content = \frac{W_3 - W_2 - W_1 \times (1 - \omega_r)}{W_1 \times \omega_r} \qquad (S1)$$

Where ω_r is the mass ratio of the rubber, calculated from the formulation of the rubber composites.

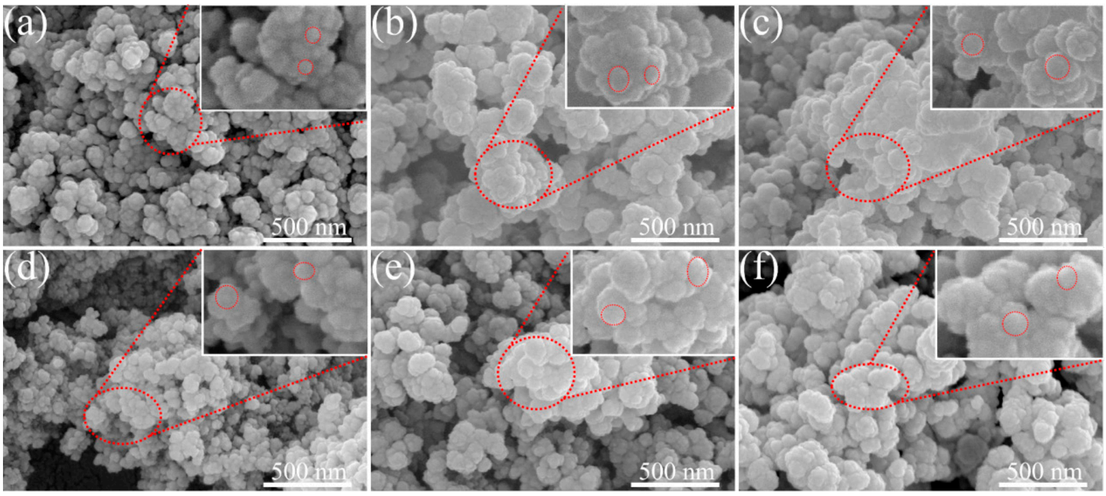

Figure S1 SEM image of (a)N115b, (b)N550b, (c)N660b, (d)N115c (e)N550c, (f)N660c.

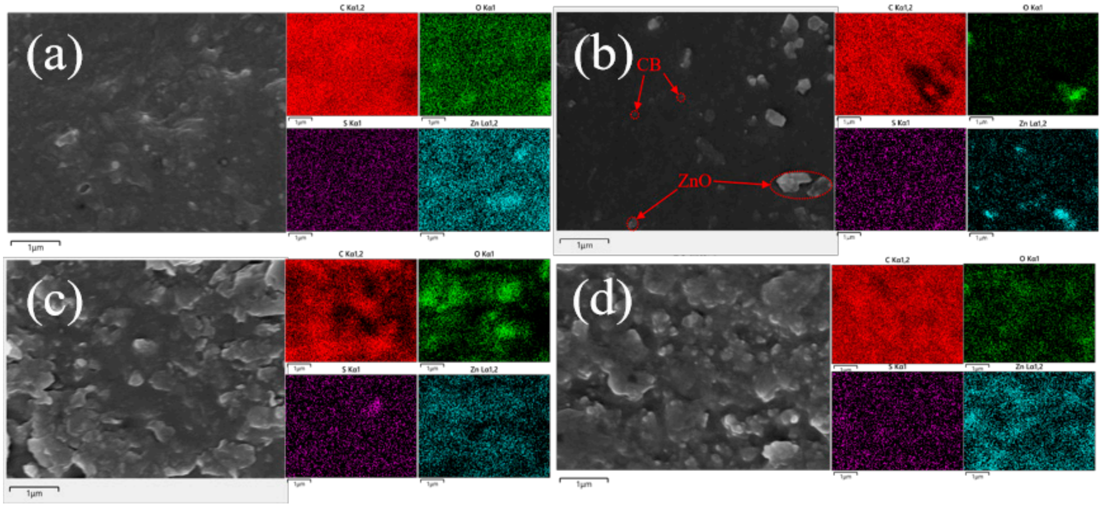

Figure S2. SEM-EDS images of (a) NR/N115a, (b) NR/330A, (c) NR/N550a, and (d) NR/N660a.

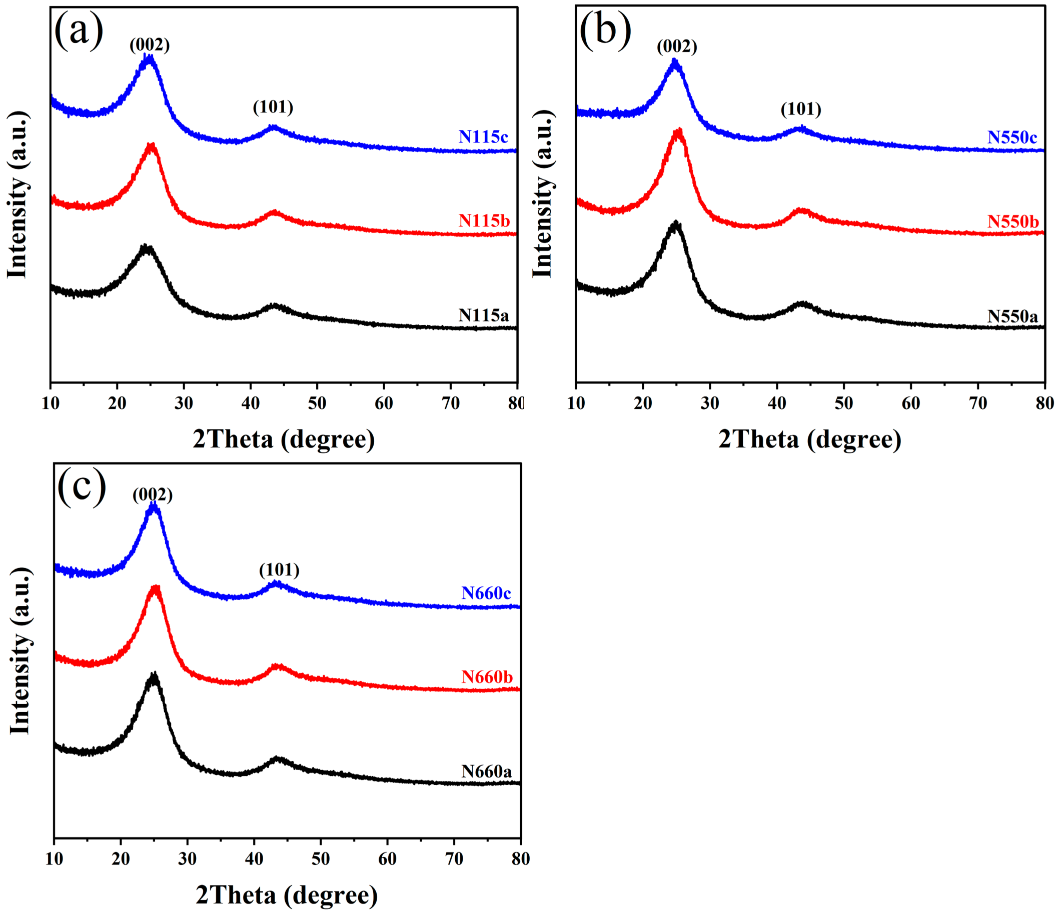

Figure S3 (a) XRD patterns of (a)N115a, N115b and N115c. (b)N550a, N550b and N550c. (c)N660a, N660b and N660c.

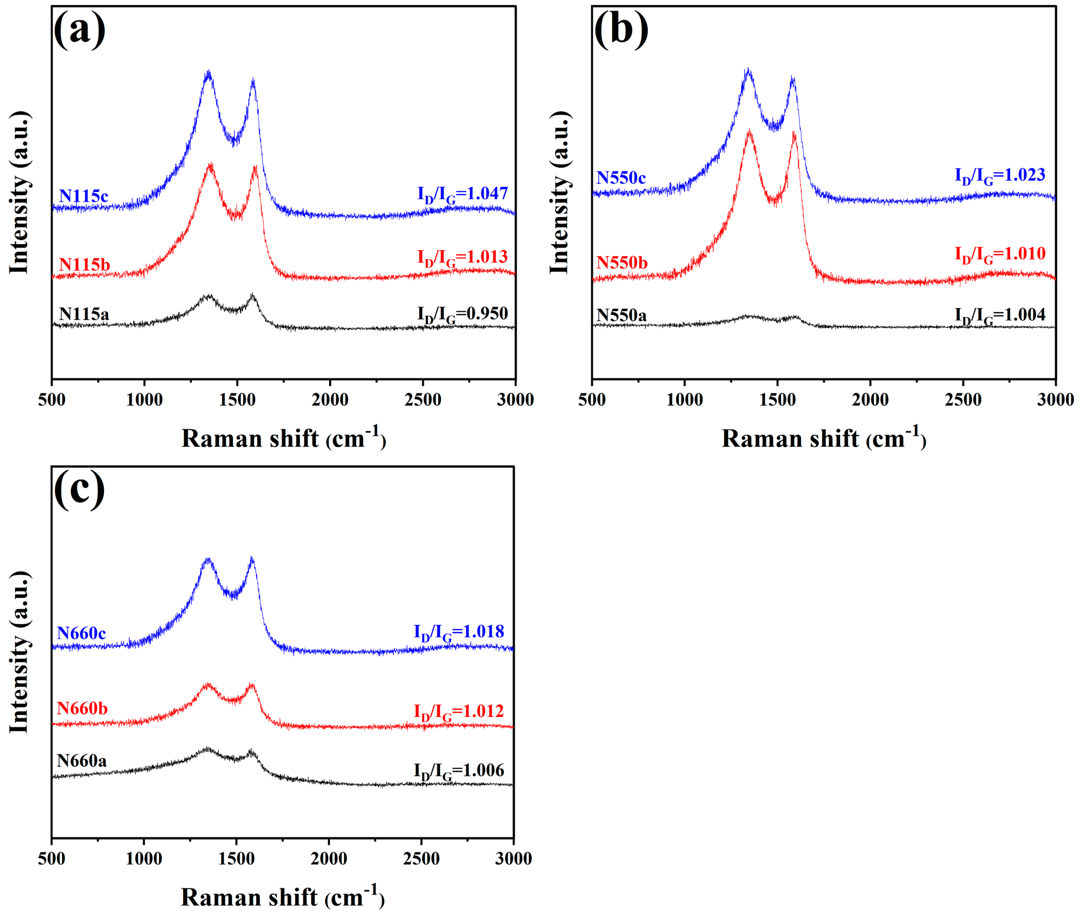

Figure S4 (a) Raman spectrums of (a)N115a, N115b and N115c. (b)N550a, N550b and N550c. (c)N660a, N660b and N660c.

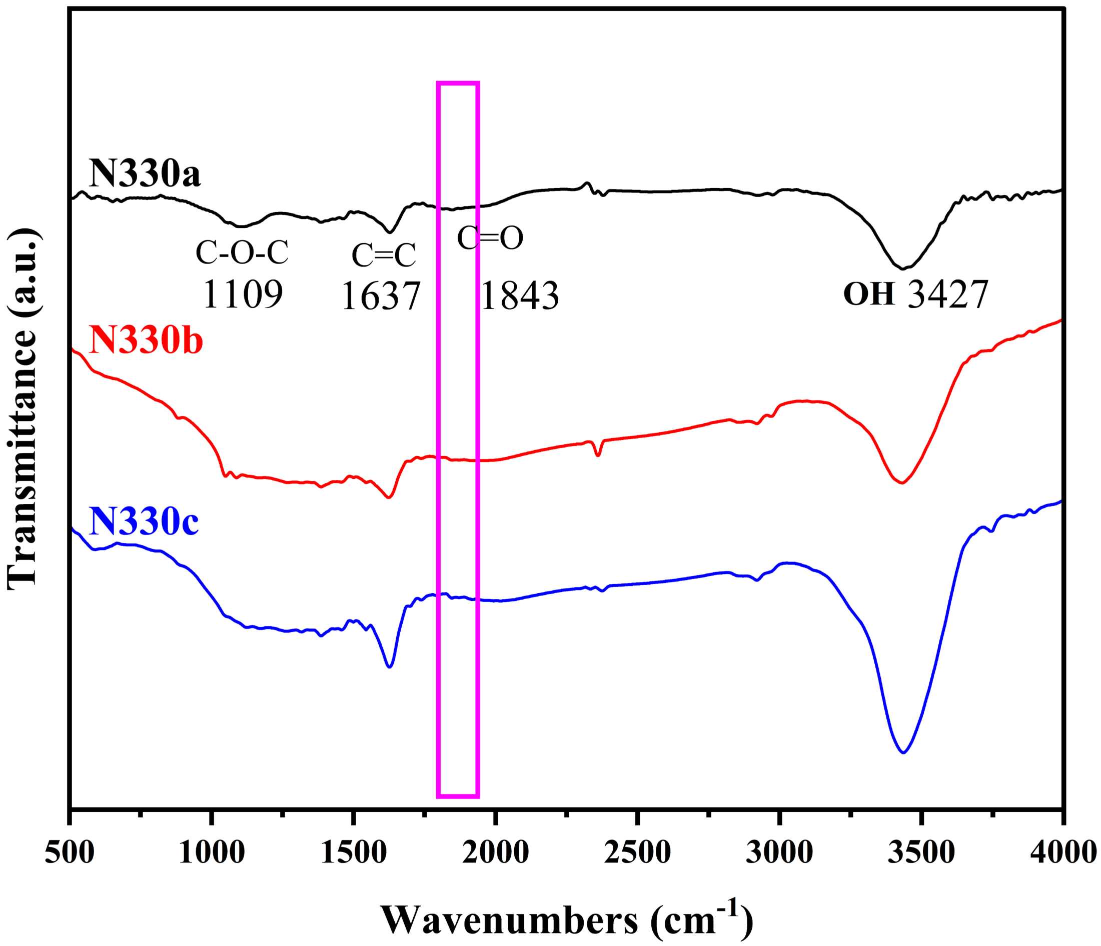

Figure S5. FTIR spectra of N330a, N330b and N330c.

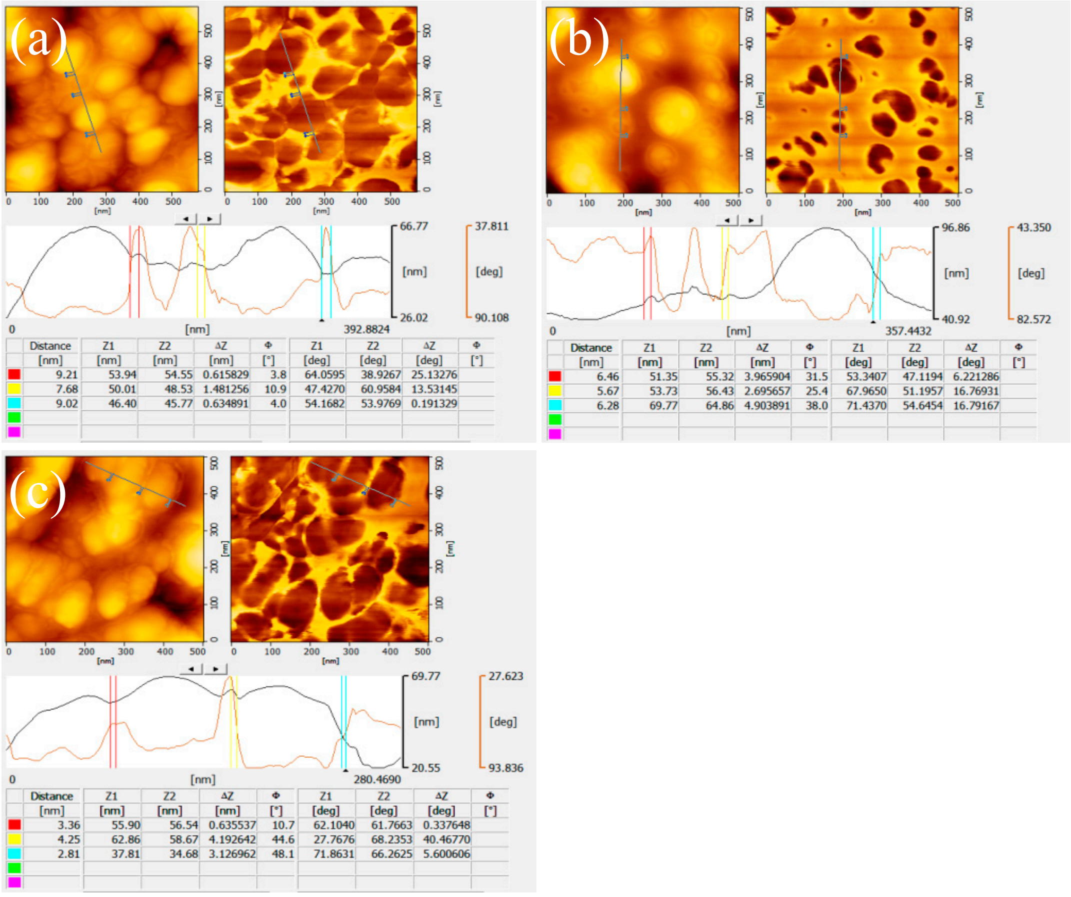

Figure S6. Fitting measurement of NR/115a, NR/N330a and NR/N550a bonding rubber.

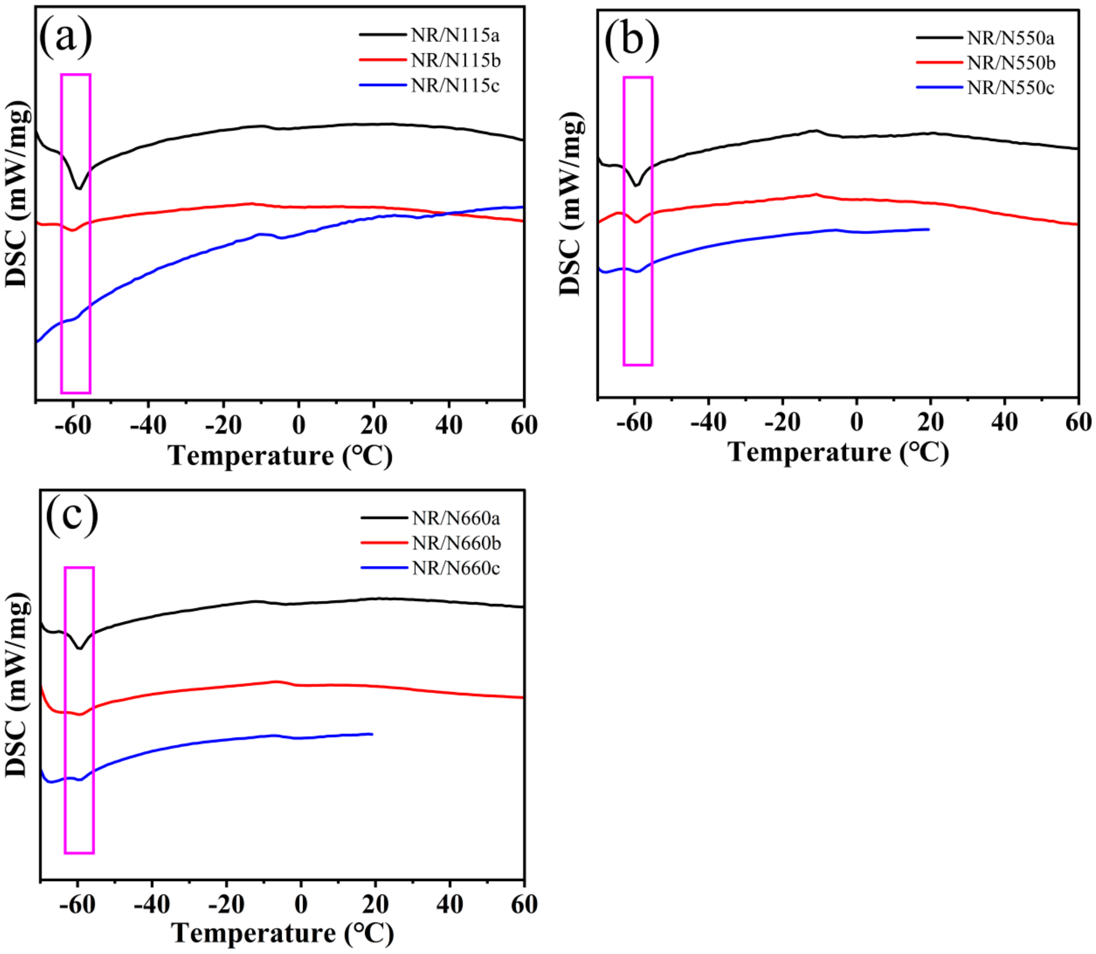

Figure S7. DSC curves of the (a) NR/N115, (b) NR/N550 and (c) NR/N660.

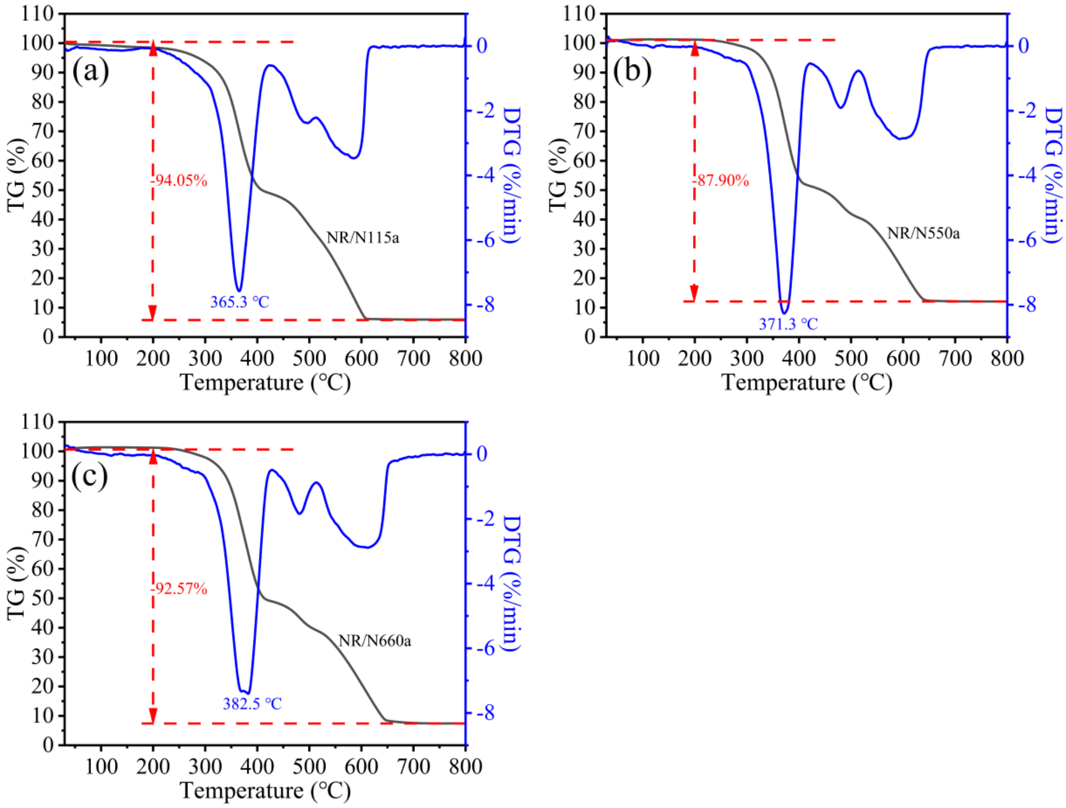

Figure S8. TG-DTG curves of the (a) NR/115a, (b) NR/550a and (c) NR/660a from 30 to 800°C.

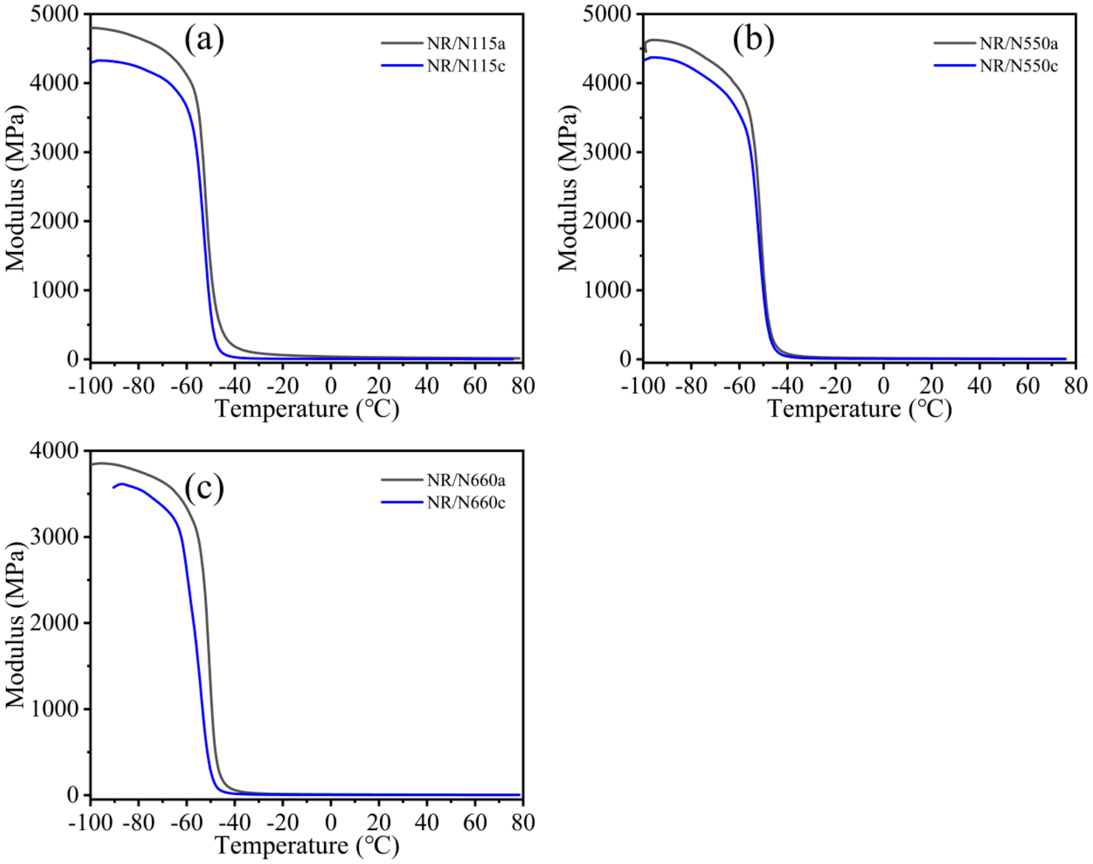

Figure S9. Storage modulus versus temperature curve of the (a) NR/115, (b) NR/550 and (c) NR/660 from -100 to 80℃.

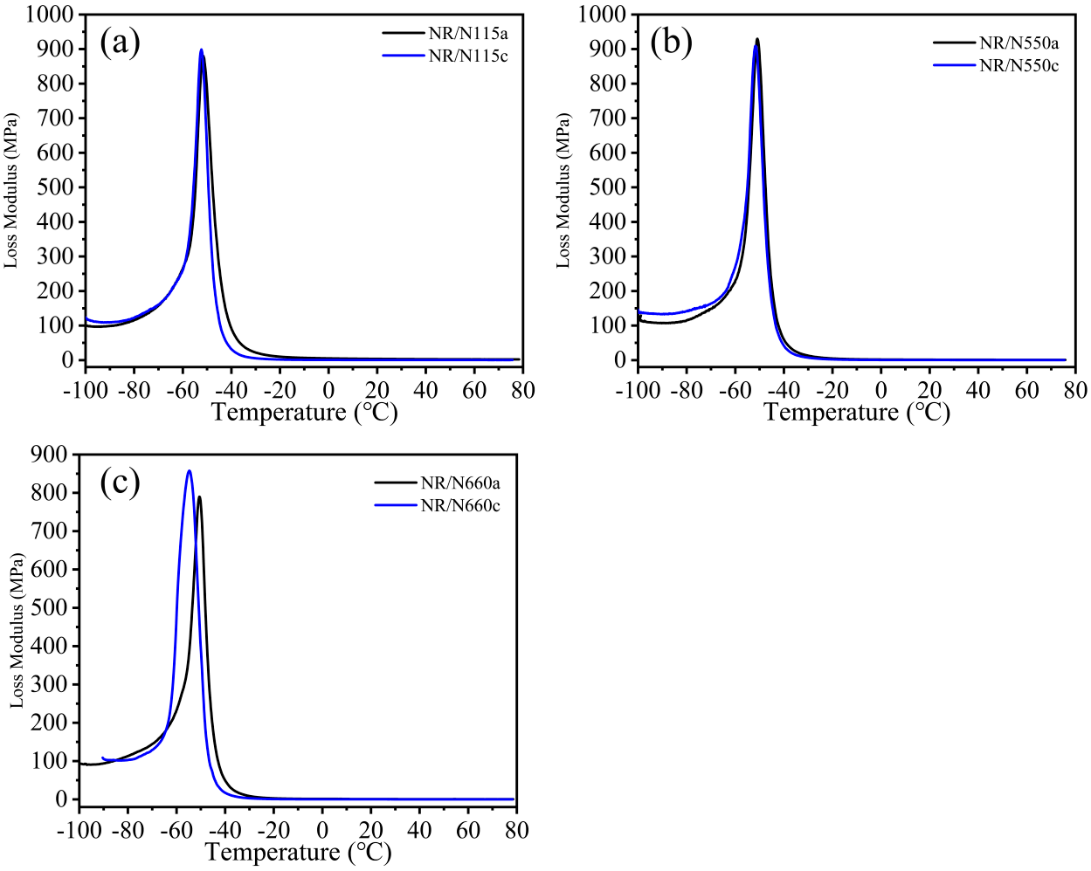

Figure S10. Loss modulus versus temperature curve of the (a) NR/115, (b) NR/550 and (c) NR/660 from -100 to 80°C.

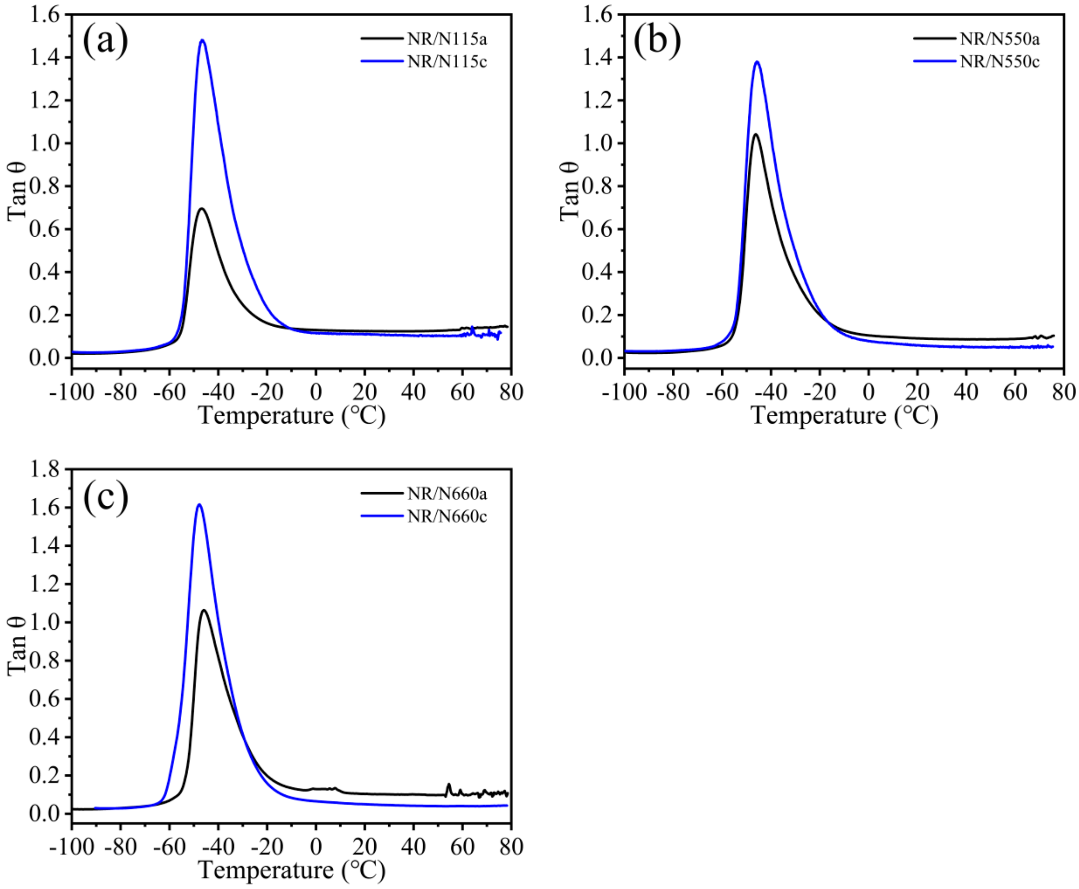

Figure S11. Loss factor versus temperature curve of the (a) NR/115, (b) NR/550 and (c) NR/660 from -100 to 80℃.

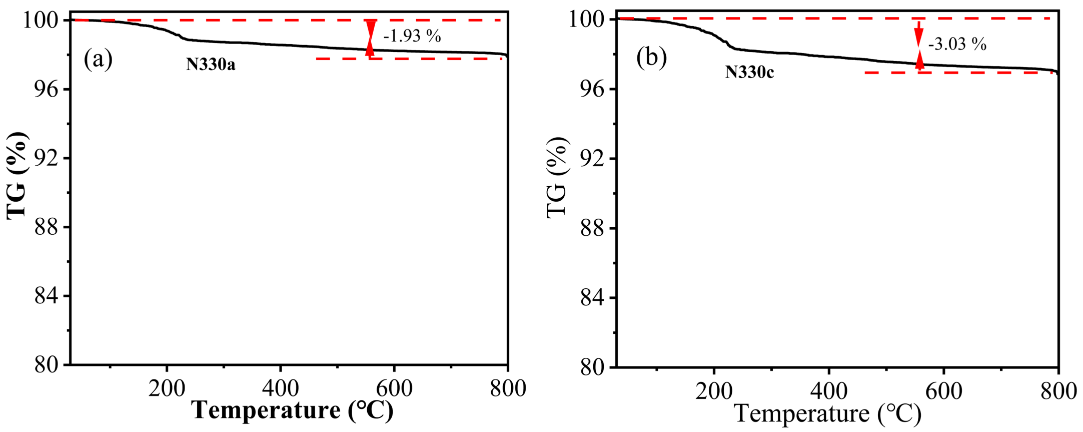

Figure S12. TG curves of the (a) N330a, and (b) N330c from 30 to 800°C, with 10 °C/min and at the maximum temperature for 30 min.
